# Screened realities: a Grounded Theory exploration of gaming disorder dynamics among Iranian male adolescents

**DOI:** 10.3389/fpsyt.2024.1357211

**Published:** 2024-05-02

**Authors:** Ali Mazaherizadeh, Zahra Taherifar, Hojjatollah Farahani, Zaheer Hussain

**Affiliations:** ^1^ Department of Psychology, University of Tehran, Tehran, Iran; ^2^ Department of Psychology, Tarbiat Modares University, Tehran, Iran; ^3^ School of Social Sciences, Nottingham Trent University, Nottingham, United Kingdom

**Keywords:** gaming disorder, internet gaming disorder, adolescents, qualitative study, grounded theory method, Iran

## Abstract

**Introduction:**

The increasing prevalence of gaming Disorder (GD) among adolescents has become a global concern. Despite the rising number of studies investigating GD, the cultural and socio-economic factors influencing GD with a qualitative approach are scarce. This study aims to explore the underlying factors, processes, and consequences of GD among Iranian male adolescents and contextual factors related to GD within Iran's unique socio-cultural and psychological tapestry.

**Methods:**

The study used a qualitative design based on the Grounded Theory Method (GTM). The researchers conducted semi-structured interviews with 13 male adolescents aged 15-18 who Dignasoed according to DSM-5 and ICD-11 criteria. The interviews were transcribed and analyzed using the GTM approach, which involves open, axial, and selective coding.

**Results:**

The study revealed nine main themes and a core category: (1) interaction seeking, (2) encounter and familiarize with games, (3) games attraction, (4) Socialization, (5) game careerism, (6) dexterity, (7) lack and compensation, (8) physical harm, territorial-cultural barrier, (9) second life, and ''life crafting'' as the core category.

**Discussion:**

The study's findings provide valuable insights into the cultural and socio-economic factors influencing GD among Iranian male adolescents. For example, Iran's economic conditions make adolescents choose gaming as their job and try to earn money in this way, which makes them more dependent on gaming. On the other hand, communities related to games play an essential role in the identity development of adolescents with GD.

## Introduction

Leisure time is increasingly spent on online video games (1). Newzoo reports 3.2 billion video game users and $200 billion in industry revenue (2). Gaming might benefit individuals (3), while a minority may develop addictions (4). The APA introduced Internet Gaming Disorder (IGD) as a tentative disorder in Section III of the DSM-5 in 2013 (5). However, there have been several arguments over the classification of gaming as a mental disorder (6, 7); WHO has included gaming disorder (GD) in the ICD-11 (8). Despite the distinct frameworks of DSM and ICD (9), both are valid and consistent (10). An essential symptom in both classifications is “loss of control over gaming” (11). In instances of discrepancies, however, ICD employs a stricter criterion, excluding individuals who do not exhibit functional impairment (12). Delphi survey experts agreed that ICD-11 criteria can diagnose GD without pathologizing normal gaming behavior (13). Threshold variations elevate IGD prevalence estimations (14). different classifications yield multiple assessment tools with various cut-off points (15), which results in disparate GD prevalence rates (16, 17). Nevertheless, recent reviews estimated GD global prevalence around 3% (18, 19); notably, male adolescents show higher GD incidence (20, 21).

Adolescents are susceptible to addictive disorders like gaming addiction (22, 23) due to their underdeveloped cognitive capacities (24, 25). Excessive gaming impacts physical activity, sleep, quality of life, academics, and social ties (26, 27). Furthermore, Excessive gaming correlates with low conscientiousness (28), self-esteem problems (29), anxiety, and depression (30, 31). Problematic gaming is associated with increased family conflict and poor relationships (32). Additionally, both the mental health of children and their parents are related to GD (33). Symptoms of GD are linked to motivations such as the avoidance of negative emotions, escapism, stress relief, and in-game social interactions (34, 35).

The Digital Games Research Center (DIREC) reports a twofold increase in Iranian digital gamers over a decade, reaching 34 million (36). Children and teenagers aged 2–17 comprise about 45% of participants. The average daily video game usage is 125 minutes for 69% of Iranian adolescents (36). Research indicates that GD prevalence among Iranian adolescents ranges from 4.2% to 17% (37–39). Gaming addiction in this group correlates with reduced physical and mental health (40).

Areshtanab et al. (41) identified a relationship between IGD and the authoritative parenting style among Iranian primary school students. However, their research needed to include the cultural depth of Iranian parenting and the significant peer influence in this age group. A recent study by Hejazifar and Livarjani (42) identified a strong link between sensation-seeking behaviors and online gaming addiction in Iranian youth. However, these sensation-seekers’ more profound personal experiences and motivations remain largely unexplored.

Iran exhibits the highest per capita opium use globally (43), which puts the Iranian society and culture in a unique situation in case of addiction (44). Previous studies have shown that variations in cultural settings may affect the patterns and correlates of online video gaming and pathological online gaming (45, 46). As GD is a novel diagnosis, it is necessary to provide a psychological understanding of this phenomenon within Iran’s social and cultural context (9, 47).

The majority of research on GD and IGD undertaken in Iran has used quantitative methods, creating a significant gap in understanding the cultural intricacies and profound personal experiences of gamers with GD. The present study adopts a qualitative design utilizing the Grounded Theory Method (GTM) to address this gap. The primary aim is to explore the underlying factors, processes, and consequences of GD among Iranian male adolescents. Additionally, this research seeks to uncover the contextual factors related to GD within Iran’s unique socio-cultural and psychological tapestry.

## Methods

### Study design

The study was based on consolidated criteria for reporting Qualitative research (COREQ) and the Journal Article Reporting Standards checklist (JARS). The research study was conducted using a qualitative method known as the grounded theory method, which is effective in describing individual behavioral patterns and lived experiences and developing theories about the major concerns in people’s lives (48). Grounded theory research primarily investigates social experiences, psychosocial processes, and the sequential steps that constitute a phenomenon or event (48, 49). The GTM is founded on symbolic interactionism, which enables people to find and comprehend meaning by interacting with others (50, 51). This approach also allows researchers to identify novel characteristics of events and provides a hypothesis that is founded on real-world experiences and is systematic (52).

The GTM stands out for its adaptable approaches to data collection, analysis, meaning extraction, and upgrading codes from the conceptual to the formal theory levels (53). The emerging theory is significant because it expands on the experience from the perspective of a particular group or environment (54). The GTM consists of theoretical sampling, continual comparative data analysis, memoing, establishing the core category, and building an exploratory theory (52). In this study, Corbin and Strauss’s Grounded Theory approach (52) was employed to examine the data and explore the underlying factors, processes, and consequences of IGD and GD among male adolescents in Iran.

### Measures

The present study utilized measurement instruments to screen participants for IGD and GD before participation. These are described below.

Farsi version of the Gaming Disorder Scale for Adolescents (GADIS-A): The scale was developed by Paschke et al. (55) as a screening tool for adolescents’ GD according to ICD-11 criteria. It has nine Likert scale items ranging from 0 (strongly disagree) to 4 (strongly agree) and an extra question assessing time criteria with options from 0 (not at all) to 3 (almost daily). Cronbach’s alpha reported 0.91 for GADIS-A. Mazaherizadeh et al. (37) adapted this scale to Persian with a Cronbach’s alpha of 0.85.

Structured Clinical Interview for Internet Gaming Disorder (SCI-IGD): Koo et al. provided SCI-IGD to evaluate IGD, considering DSM-5 criteria (56). The interview contains twelve questions evaluating nine IGD criteria. The study’s authors assessed its content validity. Seven experts from clinical and psychometric fields deemed each item necessary on a three-point scale. Based on Lawshe’s formula (57), SCI-IGD’s content validity was affirmed.

Structured Clinical Interview for DSM-5 Research Version (SCID-5-RV): The First et al. (58) tool used in this study to assess participants’ comorbid disorders. It is the most detailed version of SCID-5, encompassing numerous disorders, subtypes, severity, and periodic traits. A key feature of SCID-5-RV is its customizability for research purposes. In Iran, Mohammadkhani et al. (59) validated its content and deemed it appropriate for diagnosing disorders.

Semi-structured in-depth interview: Concerning the research literature on addictive behaviors such as gaming and gambling, researchers provided a primary interview with open-answer questions. This interview tested the pilot on three individuals who were not included among the participants. Then, the interview was fitted to be performed (the primary version of this interview is attached in the Appendix).

### Participant recruitment

The study recruited participants through SMS invitations sent to 3000 individuals from the DIREC database. Of these, around 600 expressed interest, with only three women over 18. As a result, the study was limited to male participants, which is mentioned as the study’s limitation. Of these 600 individuals, 128 were from Tehran aged 13-18, and GADIS-A was sent to them for the primary screening, which led to 28 individuals for diagnostic interview. Their diagnosis was rechecked during the interview to ensure the accuracy of their responses and prevent random answers. The participants who met the diagnosis criteria according to ICD-11 were then assessed for IGD using the SCI-IGD. Eventually, six participants were recruited. Furthermore, the study utilized snowball, purposive, and theoretical sampling. These six individuals introduced the other seven participants. The interviews were conducted online, and voice recordings and field notes were taken. The participants’ demographic information is presented in **Table 1**, with assigned numbers to protect their privacy.

**Table 1:** Demographic Information of Participants.

Participant number	Age	Grade	Birth order	Father age	Father education	Mother age	Mother education	Interview duration	Comorbid disorder(s)
1	13	Seventh	Second	45	Bachelor	45	Master’s	79	None
2	18	Twelfth	Second	53	Diploma	44	Diploma	41	Social Anxiety Disorder
3	17	Eleventh	Second	51	Middle school	45	Middle school	49	Past Major Depression Disorder
4	17	Eleventh	Second	53	Bachelor	50	Master’s	37	Persistent Depressive Disorder
5	18	Twelfth	Third	59	Middle school	46	Elementary	51	Social Anxiety Disorder
6	15	Ninth	Second	49	Elementary	47	Diploma	45	Separation Anxiety Disorder, Obsessive-Compulsive Disorder
7	18	Twelfth	Second	50	Master’s	45	Diploma	30	Generalized Anxiety Disorder, Obsessive-Compulsive Disorder
8	16	Tenth	First	42	Diploma	39	Diploma	82	Obsessive-Compulsive Disorder
9	14	Eighth	Second	55	Bachelor	40	Bachelor	36	Attention Deficit Hyperactivity Disorder
10	16	Ninth	First	45	Diploma	35	Diploma	56	Generalized Anxiety Disorder, Past Obsessive-Compulsive Disorder
11	14	Eighth	First	50	Bachelor	45	Bachelor	54	Social Anxiety Disorder
12	13	Seventh	First	52	Bachelor	48	Master’s	31	Past Generalized Anxiety Disorder
13	16	Ninth	First	42	Bachelor	39	Bachelor	43	Intermittent Explosive Disorder

### Procedure

Data was collected from June 2022 to June 2023. Before interviews, participants were briefed on the study, anonymity, and their voluntary involvement. The first author, with over four months of qualitative study training and seven years of experience as a school counselor and life skills coach for teens, conducted interviews via Skype or WhatsApp. An experienced psychologist then administered the SCID-5-RV in a separate online session. Participants were thanked with a $25 gift card. Comorbid disorders are detailed in **Table 1**.

### Ethical considerations

The study was approved by the University of Tehran’s Ethics Committee (code: IR.UT.PSYEDU.REC.1399.025). After qualifying via GADIS-A, the participant and their parents were informed about the research. Following their consent, the SCI-IGD assessment took place. Parents received further details if the criteria were met, and approved consent forms were sent for signature. Written informed consent was obtained from the minors’ legal guardians to publish any potentially identifiable data included in this paper. Participants were entitled to know their diagnosis and interview findings.

### Data analysis

All interviews were subsequently transcribed. The extensive data corpus included more than 200 pages of transcripts. Data analysis was conducted based on Strauss and Corbin’s guidelines following each interview (52). Consequently, the interview guide could be adapted to go deeper into growing concepts, and participants could be selectively chosen for further investigation. Following the transcription of the interviews, each text went through multiple readings to allow for an in-depth immersion into its contents. Data coding was performed by the first author and another expert out of the research simultaneously. The coding procedure was regularly reviewed by the research supervisors, who were the second and third authors of the study and who were university boards. The open coding process resulted in the identification of axial codes and the core category.

The data analysis process was characterized by its non-linear nature, as it involved a dynamic and continuous process that necessitated ongoing comparative analysis and subsequent modifications to the coding system. The analytical process comprises two stages, namely the paradigmatic and procedural steps. The core category emerged, and other themes were linked to this core theme. The procedure was carried out in five distinct phases: initial analysis of the data and identification of the concepts for open coding; constant comparative analyses to identify differences and similarities and provide a comprehensive description of the concepts through axial coding; analyzing the information to establish a foundation; incorporating the process into the analysis; Finally, categories were combined, linked to the core category, and the theory was refined until the theory has emerged (52). A detailed audit trail was maintained to ensure trustworthiness, and respondent validation (60) was used to verify the analysis’s credibility. Four participants were consulted for feedback, leading to grounded theory adjustments based on their insights.

## Results

**Figure 1:**
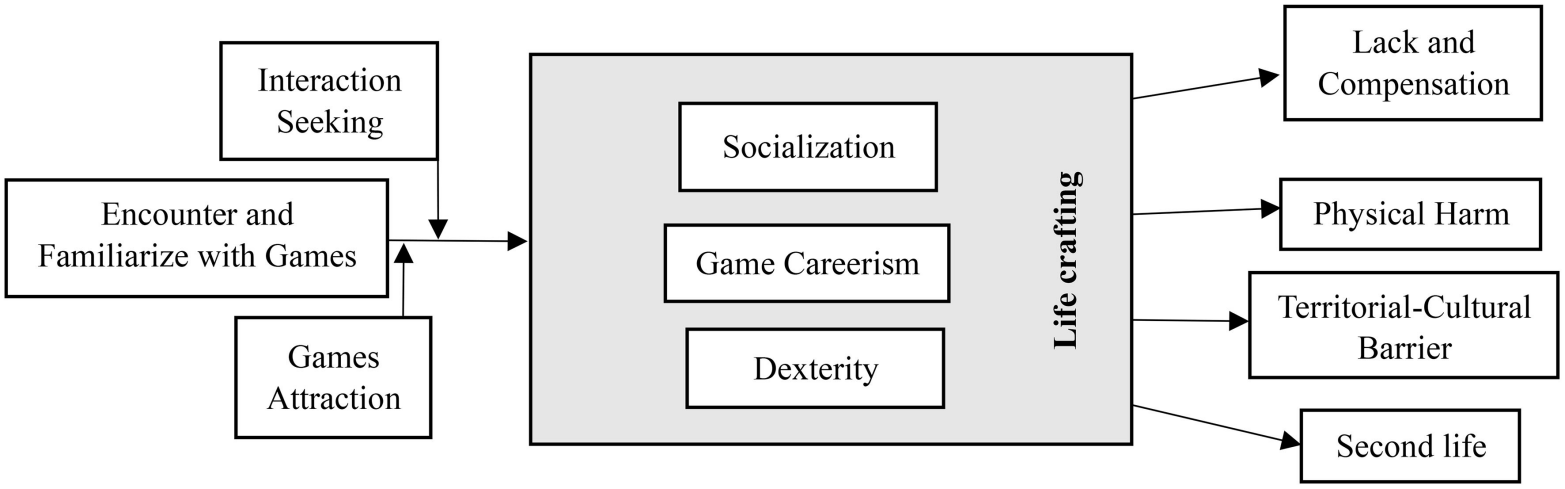
Integrated diagram of main themes around core themes.

Through data analysis, one core theme and ten main themes were discerned under the direction of Corbin and Straus (52). The themes were categorized into three main groups: Contextual Factors, Actions/Interactions, and Consequences. In the following section, we will elaborate on each theme and present a conceptual model encompassing all the themes and processes associated with developing IGD and GD in Iranian male adolescents. **Figure 1**. depicts the interplay and connections among the core themes, contextual factors, actions/interactions, and consequences.

### Core category: *Life crafting*

The core theme, “Life crafting,” delves deep into the profound influence of excessive gaming on an adolescent’s life trajectory and identity. The online gaming environment introduces teens to notable figures who have attained success, wealth, and fame, altering their perception of traditional life milestones. For instance, watching professionals on platforms like YouTube can reshape their career outlook, making gaming seem lucrative and enjoyable. This shift in perspective can sometimes lead to devaluing traditional education, seeing it as redundant or a waste of time. If gaming is not accessible, they might be reluctant to engage in academic activities due to a lack of motivation or an altered value system. Participant 1 described one of these role models,

*I was actually watching different entertaining videos on YouTube, and I came across a particular video in which a person was playing professionally, and he really attracted me to that video. In general, I realized how much money this man is making, how much he is having fun with this work, and finally, it has become his job.*

These gaming experiences also influence their future job aspirations. While some aim to pursue a gaming career, others are drawn to IT and computer-related jobs even if they are unsuccessful. Participant 8 said,

*At first, I wanted to reach a place in my field where I could become a teacher, but now that I see it is better, it is easier for me to work with a computer. I can go into interior design and decoration; working with a computer is better for me.*

The online environment fills their social interaction void and introduces them to influential figures in the gaming world. These figures often take on a guiding role, influencing their life choices and helping shape their identities. This category also highlights the importance of internet games in how teens’ identities are acquired through excessive gaming and related activities.

### Contextual factors

Contextual factors are incidents and events influencing the emergence and progression of a phenomenon, notably impacting actions/interactions. They signify individuals’ initial encounters with video games.

*Encounter and Familiarize with Games*: This theme shows the person’s exposure to games and how he learned about gaming, especially online games. Initial involvement was a significant predisposing factor in developing GD in this study. The participants’ first encounter with the game occurred between the ages of 3 and 12. Friends and social media were crucial resources for teenagers to learn about and become familiar with the games. For instance, their schoolmates introduced them to gaming or online gaming. Participant 2 described the encounter process,

*When I was between 8 and 9 years old, a friend of mine had an Android mobile phone on which he played Clash of Clans, and I loved it. At the age of nine, I received my first phone and began playing Clash of Clans.*

*Games Attraction*: Research participants played diverse game genres. The theme emphasizes games’ broad appeal, not specific genres. Some noted their game’s appeal, such as the allure of FPS games or an offline game’s engaging plot. However, the participants’ comments showed many game characteristics crucial for problematic gaming among teenagers. The game’s endless nature encourages repeated play, regardless of outcomes. Winning or losing evokes strong emotions. This allows teenagers, often engrossed in games, to experience various feelings. An engaging, endless game seems attractive and can immerse players so deeply that they lose track of time. Participant 4 explained this sensation seeking and immersion,

*People might think playing while agitated is not satisfying, but that’s not true. We may feel frustrated, but we still derive pleasure from it. This enjoyment is so intense that I become completely immersed in the game and ignore other things.*

*Interaction Seeking*: All participants mentioned a “cold” family environment, indicating a lack of emotional connection at home. Because teenagers lack relationships in the real world, they turn to online games for social interaction. This desire for communion influences their game choice, drawing them into excessive gaming. For instance, several used Zula, an Iranian FPS online game, for its communicative features. While in-game communication is vital for coordination, Iranian teenagers often extend these relationships outside games, compensating for their real-world relationship void. Participant 13 detailed the family atmosphere,

*I don’t go out of my room much. No one has anything to do with anyone in our home; only Mom and Dad sometimes speak to each other. There is no such a common connection between us. I would instead enjoy playing games with my friends because nothing is interesting about my family.*

No participant considered themselves social except one. Their underdeveloped communication skills hindered forming new relationships. Comorbid disorders with GD cannot be causally linked. However, anxiety disorders, notably social anxiety disorder, had the highest proportion of comorbidity in this study, suggesting an interaction-seeking behavior. As Participant 6 put it,

*I am timid. when I am said to buy three loaves of bread, I will turn beet red in the bakery. I don’t know why, but this is how I am.*

### Actions/interactions

Individuals use actions/interactions to engage with situations, solve problems, or navigate contexts purposefully or reactively. These acts, significant in daily life, manage or highlight phenomena. They encompass visible behaviors, emotions, beliefs, and specific responses to circumstances.

*Dexterity*: To profit and excel in a game, one must master it, altering their lifestyle to prioritize gameplay. This professional player’s lifestyle, briefly depicted in the game, overshadows other daily activities. Becoming a pro requires skill development through effort, extended play, and observing other pros. Increased gaming can also expose one to disordered gaming and its negative impacts. Participant 7 explained this professional transition,

*Someone who cares about the game becomes his priority; He follows new games everywhere and spends some time watching the games of professional players. On the other hand, a professional gamer should play at least 7-8 hours a day to maintain that level of playing.*

*Socialization*: Online games, played among real people, facilitate interaction between players. The social connections forged in games compensate for real-life relationship voids and provide a sense of belonging, which can lead to problematic gaming to maintain these connections. Many online games emphasize cooperation, where players form teams to outdo opponents collaboratively. This team dynamic offers a sense of belonging and a feeling of achievement. Such game affiliations help users feel less isolated and satisfy their social needs. Participant 8 remarked,

*Online games appeal to me because they allow you to engage with other people from all over the world. The fact that we should work well together differs from the fact that everyone can do anything they want. Furthermore, you have a team that supports you.*

*Game careerism*: Most participants were drawn to online gaming for financial gains, using unique strategies like modifying and selling user accounts. While most employed unconventional methods, only one earned through traditional gaming avenues like Esports tournaments. Two participants abstained from financial pursuits due to religious concerns. Some, like Participant 2, claimed their earnings surpassed their family’s income. Regardless of their current earnings, the prospective income remained a strong motivator, making them view gaming as a potential well-paying job with fame opportunities. This strong motivation to earn money often pushed teens to play excessively. Participant 11 put it,

*I always wanted to do something as entertainment or to earn some money or something like that. After realizing I could make money through games and become famous, I became a gamer and thought of doing online games professionally.*

### Consequences

Actions/interactions in response to a phenomenon have consequences, which can be desired or unwanted, immediate or gradual, and predictable or not. These consequences can range from minor to extensive, potentially creating a chain of events that influence future actions/interactions.

*Physical Harm*: Excessive playing causes physical injuries, which are visible consequences of GD. These bodily injuries are most likely evident to the adolescent and his family; the family’s worry is focused on these effects. The primary repercussions teenagers experienced in this study were weight gain, musculoskeletal issues, and sleep disturbance. In this regard, Participant 6 stated,

*For example, I sit down and play if I have been playing for an extended period, my eyes feel a bit heated. If you don’t have a good seat, your back will ache.*

*Second Life*: Online gaming can replace socializing, feeling accomplished, and motivating teens to work and gain money. Since he has a second life in the game, he loses essential aspects of real life. Virtual life does not have the same duties as real life. The game’s virtual world has no obligations or expectations, making it more enjoyable. Consequently, vital aspects like education may be neglected. Participant 5 stated,

*When you are playing, maybe you have a dispute with your friend in the game, but not too much. In the outside world, you have to study, take tests, or fight and argue with people who don’t like you.*

*Lack and Compensation*: The game dominates the adolescents’ lives, becoming integral to their identity. Without it, they feel a significant void, disconnected from relationships and meaningful activities. This “lack” often arises from being restricted from playing. The game also serves as a coping mechanism, helping the teenager manage negative emotions. Some even equate its importance to necessities like food and water. Participant 7 mentioned,

*I slept more that week when I was banned from playing. I used to sleep like this till midnight, wake up and watch a movie on my phone, then sleep again until morning. That week flew by, and it was really awful that I don’t want to remember it.*

*Territorial-Cultural Barrier*: Unique to Iranian society, cultural and social constraints influence adolescent gaming expectations. Families, having seen the negative impacts of gaming on their child, are often at odds when the child wants to pursue gaming as a career. This difference in views leads to conflicts between the teen and their parents. When the teenager is involved in the family’s cultural limitation conflict and endures much tension, even if parental attitudes shift, certain territorial constraints persist. In Iran, there is a pressing need for better gaming facilities. Challenges include the high costs of systems and slow internet speeds, which are crucial for online gamers. According to participant 12,

*Look, this is Iran, and there are many limits; for example, the internet connection is so slow that you can’t even access WhatsApp with it, or I purchased a handheld device that can’t play many games, so you can’t select gaming as a job.*

## Discussion

This research aimed to cultivate a comprehension of GD and IGD within male adolescents in the context of Iranian culture. To the best of the author’s knowledge, this study is the first investigation into the phenomenon of IGD and GD amongst Iranian gamers utilizing a qualitative research methodology and the grounded theory approach. This study has discovered new and culturally influenced observations in GD, which will be discussed further.

The study identifies three subcategories in “Encounter and Familiarize with Games”: age of initiation, social media, and peer influence. Early exposure is linked to problematic internet use (61) and IGD (62), with peer influence, often through game invitations, correlating with gaming intensity (63, 64). Today’s “digital natives” (65) are immersed in the digital world from a young age (66), frequently encountering online games via social media.

This study suggests that certain game features may contribute to Game Disorder (GD), including endless nature, complexity, first-person shooter, fantasy graphics, strategy, immersion, and role-playing elements (67, 68) a wide range of games could lead to GD regardless of genre (28). Sensation-seeking, which encompasses positive and negative emotions experienced during gaming, is linked to GD (69). Competitive and interactive elements in games, fostering player competition and a sense of achievement, are shown to excite players and are associated with GD (9). It seems that games capable of providing these immersive and competitive experiences could potentially possess addictive qualities.

In the context of GD, interaction-seeking involves two subcategories: emotional fulfillment and social anxiety. Adolescents with poor family attachments, a known risk factor for GD (27, 70), often turn to online gaming for communication needs. These findings align with Salehi et al.’s study (71) which indicated a negative correlation between secure attachment and IGD among Iranian adolescents. Additionally, social anxiety plays a dual role. It’s seen as an inherent trait in introverts, linked to IGD (9, 27), and as a disorder where virtual spaces provide a safer environment for socializing, especially beneficial for those with social anxiety, as it’s less stressful than face-to-face interactions (72).

The socialization category in online gaming, crucial for adolescents seeking connections and belonging (73), is a response to interaction seeking and a key motivator for those with disordered gaming symptoms (74). Its role in excessive gaming is notable (28). However, a study by Rafiemanesh et al. (75) showed that Iranian university students prioritize recreation over socialization, possibly due to their communication needs being met within the university environment, unlike adolescents. Consequently, gaming communities become vital for teenagers, offering recognition and a sense of authenticity. Vilasís-Pamos et al. (76) identified five gamer categories, with two groups, celebrity-platform gamers, and professional gamers, aiming for fame, fans, and professional status. This desire for recognition and achievement in gaming communities is a primary motivation for participants in this study.

The second life means a person provides a complete life in the game’s virtual world. Online games allow individuals to meet real-life needs (70) (e.g., belonging, achievement, and connection with others). This virtual life in the game space separates the teenager from the difficulties and responsibilities of real life, causing him to ignore one of his most important personal responsibilities which is education. Previous research suggests academic failure as a consequence of GD (21).

In this study, physical harm indicates damage to the body, such as inactivity, weight gain, and skeletal-muscular problems, which are also associated with excessive playing, according to previous studies (16). As previously stated, sleeping problems are a significant side effect of GD (26). However, Due to internet restrictions in Iran, teens opt to play games at night to obtain better quality internet, which worsens the difficulty of sleeping among Iranian teenagers suffering from GD.

The SCID-5-RV results revealed that all participants -except one- had at least one clinical diagnosis besides GD and IGD, with significant comorbidity of psychological disorders, especially anxiety disorders, suggesting excessive gaming as a distress-related coping mechanism (77). This supports the study’s model linking the need for communication and relationship formation in Iranian adolescents with GD and IGD. Comorbidity with disorders like anxiety, depression, and obsessive-compulsive disorder aligns with prior research (31, 72). While intermittent explosive disorder (IED) isn’t typically comorbid with GD, its associated impulsivity, a common symptom in IED and ADHD (78, 79), is a known risk factor for GD (16, 80). Further research is needed in Iran to explore comorbid disorders with GD.

In IGD research, escape, or mood modification is akin to gaming to change mental state in this study. Montag et al. (9) identified escape motivation as a critical predictor of GD. Another study found that individuals with IGD use gaming to alleviate negative emotions linked to disorders like major depression and dysthymia (81). This trend is evident in our study, where most participants with psychological disorders used gaming to improve their mental state, aligning with Davis’s model (77) that suggests underlying psychological issues drive the problematic use of games for mood regulation.

DSM-5 characterizes withdrawal symptoms in gaming as arising from an inability to access games (82). In this study, such withdrawal, termed ‘lack’ following game deprivation, was prevalent among participants. This finding varies from Holm et al. (83), who reported withdrawal symptoms in 59% of their sample, and Yen et al. (84), who observed that 85% of their IGD participants experienced symptoms like an urge to play and emotional distress, which gaming alleviated. These results are consistent with our study, indicating that adolescents use gaming to compensate for their experienced lack.

The “life crafting” category highlights the significant impact of GD on the lives and worldviews of affected youth. During the interview, the researcher was incredibly driven by the participants’ passionate descriptions of the game and related events. Memos show that the interviewer desired to experience the discussed online games personally after some interviews. Life crafting was chosen as the core theme because of this profound experience and continual comparisons of concepts and categories. This choice reflects the profound influence of gaming, particularly during adolescence, a crucial stage for identity development (85). The influence of group norms and virtual communities on behavior and social identity is notable (86, 87), with some games enabling players to craft alternative identities (88).

In this study, participants’ identities were deeply intertwined with gaming, shaping their attitudes towards school, careers, and relationships, aligning with Bacchini et al. (88), who found MMORPG players often neglect real-world responsibilities and question their life choices. This research uniquely explores online gaming’s impact on various identity dimensions, revealing that games significantly influence adolescents’ identity and life direction. Participants, having played games since early childhood without alternative identity exploration sources, often model their identities on prominent figures in gaming communities. This aligns with social identity theory (89), which suggests connections to social referents shape self-concept. Future research should investigate the impact of these figures (e.g., professional gamers, YouTubers, and Streamers) on adolescents’ identity and gaming behavior.

Teenagers’ career choices are shaped by their gaming-influenced identity in three ways. First, those who don’t see themselves as superior gamers often pivot towards computer-related careers, perceiving them as convenient, flexible, and profitable. Second, the ‘game careerism’ category reflects a desire among adolescents to pursue game-related professions. This aligns with the growing popularity of professional gaming and e-sports among youth (90). Finally, this study reveals that beyond traditional gaming careers like streaming, YouTubing, or professional gaming, some participants consider ancillary roles, such as editing videos for YouTubers, viable career options. Participants in this study chose eSports careers based on factors like convenience, flexibility, and entertainment, which is consistent with Said et al.’s findings (91). Additionally, the potential for monetary gain was a key motivator. With technology’s expected growth, the demand for computer-related jobs is likely to increase, influencing young people’s career choices due to the prevalence of computers, games, and media in everyday life. Longitudinal research is needed to assess the impact of GD on adolescents’ career paths and its broader individual and social effects.

Financial incentives were a key motivator for nearly all participants in this study, linked to the increased severity of IGD symptoms as shown in previous research (92). However, Iranian adolescents aspiring to become professional gamers face cultural-territorial challenges, including high costs of gaming systems, poor internet quality, and sanctions hindering participation in e-sports events. Culturally, many parents in Iran, similar to findings by Jiow et al. (93), do not recognize pro-gaming and YouTube careers as legitimate, often expressing concerns about time management and academic neglect. This reflects a parental perspective similar to that in Iran, though children’s attitudes may differ.

Despite cultural-territorial barriers, Iranian adolescents continue to pursue gaming as a source of income and career choice. They engage in activities like enhancing and selling gaming accounts or trading in-game items for profit. Iran’s challenging economic conditions, marked by a limited job market, inflation, and financial instability (94, 95), drive adolescents towards alternative income sources like gaming. The global nature of the gaming sector offers access to international markets and the potential to earn more valuable foreign currencies amidst the national economic crisis. This unique economic context in Iran may fuel the trend of adolescents seeking income through gaming, a notable difference from their peers in more stable economies. Further research is needed to understand the relationship between economic conditions and the pursuit of gaming-related income.

In gaming, individuals find a sense of belonging, achievement, and potential income, leading them to improve their skills continually. The ‘dexterity’ category highlights the belief in the importance of skill enhancement, which is achievable only through frequent play. Professional gamers facing high-performance demands show higher rates of disordered gaming (96). Despite its slim chances of success, the pursuit of professional gaming may increase the risk of excessive gaming among players (90).

## Limitations

The present study has limitations that need careful consideration. Primarily, the necessity for remote data collection, with interviews conducted online, might have influenced the depth of information obtained. This method of data gathering could potentially impact the accuracy of participants’ self-reported data.

Furthermore, the coexistence of IGD and GD within the same study sample raises the possibility of symptom severity overlap, potentially confounding the differentiation between the two conditions and affecting the conclusions’ precision. Additionally, the recruitment of some participants through DIREC might have introduced bias, as individuals who actively chose to participate could represent a more engaged subgroup with distinct experiences, potentially affecting the findings. Furthermore, the study was limited to male participants due to the lack of access to female participants, resulting in limited gender diversity and potential bias in findings.

## Conclusion

This pioneering qualitative study offers an understanding of GD among Iranian male adolescents, unveiling its cultural nuances and multifaceted nature. The research highlights the significance of early exposure, social media, and peer influence in driving excessive gaming while delving into the psychosocial dimensions of interaction seeking, family attachment, and social anxiety. The concept of “life crafting” reveals how gaming shapes adolescents’ identities and career aspirations. Furthermore, the clash between aspirations for professional gaming careers underscores the need for culturally sensitive interventions. By contextualizing GD within the Iranian context, this study provides valuable insights for tailored strategies and opens avenues for cross-cultural exploration of the phenomenon.

## Data availability statement

The raw data supporting the conclusions of this article will be made available by the authors, without undue reservation.

## Ethics statement

The studies involving humans were approved by University of Tehran’s Ethics Committee (code: IR.UT.PSYEDU.REC.1399.025). The studies were conducted in accordance with the local legislation and institutional requirements. Written informed consent for participation in this study was provided by the participants’ legal guardians/next of kin. Written informed consent was obtained from the minors’ legal guardians for the publication of any potentially identifiable data included in this paper.

## Author contributions

AM: Writing – review & editing, Writing – original draft, Methodology, Investigation. ZT: Writing – review & editing, Supervision, Methodology. HF: Writing – review & editing, Supervision, Methodology, Data curation. ZH: Writing – review & editing, Supervision.

## Appendix

**Table:**

Main questions	Following questions
What’s your idea about internet gaming?	- How did you get acquainted with online games? - When did you start? (How long have you been playing)? - How often do you play? - When do you usually play during the day? - How much time do you spend each time you play?
Tell me about the games you play.	- What games do you play? - Do you play one specific game or play various games? Have you experienced other games? - What is attractive about this game rather than other games? Why do you prefer this game? - Do you practice your game when you are offline? - Do you spend money for gaming? How much? - Have you ever been in trouble for spending a lot money? - What is your rank? Does your rank matter to you? Why?
What is your motivation/reason for playing?	- When do you go to play usually? When do you need more playing? - What makes you start playing? (thoughts, feelings, special moments)
Tell me about your experience when you are playing.	- What do you feel when you are playing? - How does this type of game make you feel compared to than other games? - What is your reaction when you fail? What about winning? - What is enjoyable about gaming? - Do you feel like that something is preventing you to stop gaming? Can you explain it?
Tell me about the people you play with?	- Who are you playing with? Are they your friends or meet them in game space? - Do you care who are your teammates? Do you care about real personality of your teammates? - How is your relationship with your teammates? Do you have relations out of game space?
Does playing affected your life?	- Relationships, family, friends - lifestyle, goals - school, home works - is there anything else you want to do?
Can you explain me a regular day of your life?	- How do you spend your leisure time? Is it different now than before familiarize with gaming? – What is enjoyable about these leisure activities?
Tell me about your family?	- Parents relationship with each other - Parents relationship with other sibling - Parents attitude toward gaming - Your relationship with sibling - family atmosphere in general - Can you describe your parents?
How do you describe yourself?	- How does your family and friends describe you? - How did teachers describe you? - How many close friends do you have? Do others consider you a social person?
Are you active in social networks?	- Which networks or applications? - how much time do you spend on them? - Do you post about your gaming through social networks? - Does it matter to you that your gaming activities seen by others on social network?
Have you ever been banned from playing?	- How was your experience during that period? - family, therapy or….
What do you think about earning money from gaming?	- have you ever been made money from gaming? How much? - How did you made this money?
Is there anything that I didn’t ask and you want to explain?	
